# Beneficial Actions of *Orostachys japonica* and Its Compounds against Tumors via MAPK Signaling Pathways

**DOI:** 10.3390/nu13020555

**Published:** 2021-02-08

**Authors:** Soyoung Hur, Eungyeong Jang, Jang-Hoon Lee

**Affiliations:** 1 Department of Clinical Korean Medicine, Graduate School, Kyung Hee University, Seoul 02447, Korea; vicalcandy@naver.com; 2 Department of Internal Medicine, College of Korean Medicine, Kyung Hee University, Seoul 02447, Korea; obliviona79@naver.com; 3 Department of Internal Medicine, Kyung Hee University Korean Medicine Hospital, Seoul 02447, Korea

**Keywords:** *Orostachys japonica*, kaempferol, quercetin, tumor

## Abstract

Tumors are one of the most life-threatening diseases, and a variety of cancer treatment options have been continuously introduced in order to overcome cancer and improve conventional therapy. *Orostachys japonica* (*O. japonica*), which is a perennial plant belonging to the genus *Orostachys* of the Crassulaceae family, has been revealed to exhibit pharmacological properties against various tumors in numerous studies. The present review aimed to discuss the biological actions and underlying molecular mechanisms of *O. japonica* and its representative compounds—kaempferol and quercetin—against tumors. *O. japonica* reportedly has antiproliferative, anti-angiogenic, and antimetastatic activities against various types of malignant tumors through the induction of apoptosis and cell cycle arrest, a blockade of downstream vascular endothelial growth factor (VEGF)-VEGFR2 pathways, and the regulation of epithelial-to-mesenchymal transition. In addition, emerging studies have highlighted the antitumor efficacy of kaempferol and quercetin. Interestingly, it was found that alterations of the mitogen-activated protein kinase (MAPK) signaling cascades are involved in the pivotal mechanisms of the antitumor effects of *O. japonica* and its two compounds against cancer cell overgrowth, angiogenesis, and metastasis. In summary, *O. japonica* could be considered a preventive and therapeutic medicinal plant which exhibits antitumor actions by reversing altered patterns of MAPK cascades, and kaempferol and quercetin might be potential components that can contribute to the efficacy and underlying mechanism of *O. japonica*.

## 1. Introduction

Despite recent advances in diagnosis and treatment, cancer remains one of the most life-threatening diseases. According to the 2016 World Health Organization (WHO) databases, tumors impose the heaviest disease burden and are responsible for 8.97 million deaths worldwide annually [1]. Notably, it is predicted that the contemporary prevalent trend of cancer incidence, morbidity, and mortality will maintain its incremental increase over the next four decades [2]. As part of the ongoing fight against cancer, various types of treatment strategies in oncology are evolving. Since mechlorethamine was first approved in 1949 by the US Food and Drug Administration (FDA) for treating lung cancer, leukemia, and lymphoma [3], more than 100 chemotherapeutic agents that kill malignant cells have been used [4]. However, cytotoxic drug resistance, low responses, and adverse effects are still major impediments to patients that undergo chemotherapy.

New generation anticancer strategies, such as target-specific drugs, hormonal agents, immunotherapies, and combination cancer therapy, have been introduced to effectively inhibit and prevent the overgrowth and spread of tumor cells by increasing the drug sensitivity, while producing minimal side effects [5]. However, as tumors have various forms and stages, not all patients with malignancy can use and benefit from new anticancer regimens. Meanwhile, cancer cells adapt better to novel treatments, presenting a large challenge to the clinical management of advanced neoplastic diseases. Improved drugs also fail to completely eliminate cancer cells without toxicity, which leads to recurrence, aggravation, and metastasis [6]. Accordingly, there is a significant demand for safe and multitargeted innovative treatments to overcome stubborn tumors. In addition to clearing cancers, cancer treatments aim to alleviate clinical manifestation, control metastasis, and improve the quality of life of patients.

To develop new effective anticancer drugs, researchers and clinicians are now considering medicinal plants. The development of novel drugs requires a long period of time (average of 13 years), and the process of their development from bench to bedside has a high cost [7]; the use of herbs can save time and expenses required for drug discovery. Moreover, numerous experimental and clinical studies have supported the safety and efficacy of herbal medicine in cancer treatment [8]. In fact, 146 of the 174 chemotherapeutics in the anticancer drugs market from 1981–2014 were related to herbal plants [9]. Therefore, herbal products can drive growth in the anticancer drug market.

*Orostachys japonica* A. Berger (*O. japonica*), which is a perennial herb belonging to the family Crassulaceae, is called Wasong (roof tiles-pine) in China and Korea; the name comes from its tendency to grow on roof tiles or rocks in a shape resembling pine leaves or flowers [10]. After the removal of *O. japonica* roots, the dried aerial sections of plants have long been used as a traditional remedy to alleviate diverse symptoms, such as fever, inflammation, bleeding, intoxication, and hemostasis. In consideration of the ongoing research focusing on *O. japonica*, its extract has been revealed to possess a wide range of biological actions, including antiproliferative, anti-angiogenic, and antimetastatic properties against tumors. Additionally, kaempferol and quercetin, which are representative flavonoids from *O. japonica* extracts, are well-known as promising anticancer compounds.

To the best of our knowledge, a literature review focusing on the beneficial role of *O. japonica* against various malignancies has not yet been performed, despite accumulating preclinical evidence. Therefore, in order to provide useful information on how *O. japonica* exerts anticancer effects, we reviewed available preclinical studies of *O. japonica* and its compounds using cancer-related models. We herein attempt to document its promising efficacy and the mechanisms involved in its activities, which may demonstrate its medical value as an anticancer drug.

## 2. Phytochemistry of *O. japonica*

Ingredient analysis studies on quality control and standardization of the chemical constituents obtained from *O. japonica* are largely carried out in Korea. According to studies published until now, *O. japonica* has been reported to contain sterols, flavonoids, phenolic acids, and triterpenoids [11]. Twelve flavonoid compounds have been separated and identified from *O. japonica*, including kaempferol, quercetin, astragalin, quercitrin, isoquercitrin, cynaroside, afzelin, 3-o-a-L-rhamnosyl-7-o-13-d-glucosyl kaempferol, 3,7-di-o-13-d-glucosyl kaempferol, kaempferol 3-o-β-d-glucoside, kaempferol 3-o-β-d-galactoside, and quercetin 3-o-β-d-glucoside [12,13]. In addition, (−)-epicatechin, (−)-epicatechin 3,5-digallate, (−)-epicatechin 3-gallate, and (−)-epicatechin 5-gallate, belonging to the flavonol subgroup of flavonoids, were isolated from the aerial part of *O. japonica*. The phenolic acids gallic acid, 4-hydroxybenzoic acid, 3,4-dihydroxybenzoic acid, and methyl gallate were also purified from *O. japonica* as phenolic compounds [13]. Taraxerone of 3-oxo-triterpene, belonging to the triterpenoids group, and steroids such as stigmast-4-ene-3-one and ergost-4-ene-3-one of 3-oxo-steroid were also characterized from whole *O. japonica* plants using spectral analysis [14]. Therefore, previous studies have revealed the presence of about 34 kinds of chemical compounds, which were identified via phytochemical research on *O. japonica*.

In the 2005 Chinese Pharmacopeia, Wasong was introduced as the aerial part of *Orostachys fimbriatus* (Turcz.). Berger and its dry herb should contain quercetin (C_15_H_10_O_7_) and kaempferol (C_15_H_10_O_6_) contents of more than 0.020% [15]. The origin and purity test standards of *O. japonica* are described in the Korean Herbal Pharmacopeia published in 2020, but there is no record showing the data of the confirmatory test using its indicator compounds [16]. However, using column chromatography from the ethyl acetate fraction of *O. japonica*, the contents of kaempferol, quercetin, and gallic acid were quantified as 6.81%, 5.08%, and 4.24%, respectively [17]. Moreover, a recent study protocol for a clinical trial showed that an *O. japonica* water extract should contain 1% or more gallic acid (C_7_H_6_O_5_) as an index compound [18].

Therefore, it is possible that the main constituents for the quality control of *O. japonica* will be those of kaempferol, quercetin, and gallic acid. For the standardization of *O. japonica* in a further study, more data will need to be gathered on the content standards of active ingredients, the optimal extraction methods, and the establishment of an analysis method to separate key components.

## 3. Anticancer Properties of *O. japonica*

### 3.1. Antiproliferative Effect

Sustained proliferative signaling is regarded as a representative biological hallmark of cancer [19]. Therapeutic strategies that decrease the proliferation rate of cancer can be useful for regulating cell death that is uncontrolled in cancer. For this reason, cytotoxic nucleoside analogues were used as the first chemotherapeutic agents to manage cancer [20]. Therefore, in order to present preclinical evidence on the anticancer effects of *O. japonica*, it is important to investigate whether its extract suppresses cell proliferation in tumor cells.

The ability of *O. japonica* to inhibit the growth and proliferation of a variety of cancer cells has been determined by analyzing its effects on cell survival and tumor size. To assess the cell viability and proliferation of tumor cells in the presence of *O. japonica*, cell viability assays such as 3-(4,5-dimethylthiazol-2-yl)-2,5 diphenyl tetrazolium bromide (MTT), 3-(4,5-Dimethylthiazol-2-yl)-5-(3-carboxymethoxyphenyl)-2-(4-sulfophenyl)-2H-tetrazolium (MTS), phenazine methosulphate (PMS)/MTS, water-soluble tetrazolium salts (WST), and sulforhodamine B (SRB) assays; cell counting assays using microscopy or dyes; and a [^3^H]-thymidine incorporation assay based on DNA synthesis, were performed. In a rodent model injected with SW480 colon cancer cells, the actual weight and volume of the excised tumor were calculated; a reduction in tumor size indicates the inhibition of tumor cell proliferation.

*O. japonica* treatment has been found to exhibit significant cytotoxicity against gastric (AGS and KATO III), liver (HepG2 and Hep3B), bile duct (SNU-1079), pancreas (PANC-1), lung (Calu-6 and A549), colon (HT-29 and SW480), melanoma (A375), leukemia (HL60, THP-1, U937, L1210, and K562), prostate (RC-58T/h/SA#4 and LnCaP), cervix (HeLa), ovary (NIH:OVCAR-3), and breast (MDA-MB-231 and MCF-7) cancer cells, as demonstrated by its ability to lower the percentage of cell viability compared to controls. In addition, viable cancer cell lines tend to grow abnormally by attaching to the well. However, a large portion of AGS, KATO III, HepG2, Hep3B, SW480, and RC-58T/h/SA#4 tumor cells were detached from the well and floating in the culture containing *O. japonica* extract. In particular, when using a trypan blue assay to dye damaged membranes, significant increases in the number of non-viable floating cells in SW480 and RC-58T/h/SA#4 cell lines were observed. Furthermore, the addition of 10 and 100 μg/mL *O. japonica* water extract induced a significant reduction in DNA synthesis in HepG2 and K562 cells, respectively, using a [^3^H]- thymidine incorporation assay. These results suggest that the *O. japonica* extract suppressed the proliferation of DNA-synthesizing cancer cells [21]. Therefore, stimulation with *O. japonica* extract inhibited the overgrowth of a large number of cancer cells (Table 1).

In view of the half maximal inhibitory concentration (IC_50_)values of *O. japonica* in tumor cells, its extract yielded 50% cancer cell death in a wide range of 50–997.4 μg/mL in AGS, Calu-6, MCF-7, PANC-1, and U937 cells. The lowest IC_50_ value calculated was 50 μg/mL of the ethyl acetate fraction of the 95% ethanol extract from *O. japonica*, causing half of the PANC-1 pancreatic cancer cells to die [22]. This result implies that promising bioactive compounds that are responsible for the antiproliferative effects of *O. japonica* against PANC-1 cells may be obtained from the ethyl acetate fraction of its 95% ethanol extract. The ethyl acetate fraction of *O. japonica* has also shown strong cytotoxic effects against MDA-MB-231 and OVCAR-3 cells at relatively low concentrations of 60 and 50 μg/mL, respectively. However, when assessed against SNU-1079 cholangiocarcinoma cells, the water extract of *O. japonica* was proven to possess stronger cytotoxic effects than those induced by its 50% ethanol extract. The methanol extract of *O. japonica* effectively inhibited the abnormal growth of RC-58T/h/SA#4 prostate cancer cells induced by environmental hormones (dioxin and bisphenol A), as well as against prostate cancer without these hormones. Accordingly, optimal extraction solvents and efficient doses to maximize the antiproliferative effects of *O. japonica* need to be determined, depending on the type of tumor (Table 1).

Interestingly, *O. japonica* even displayed significant cytotoxicity against A375 melanoma cells that are highly resistant to chemotherapy [23] (Table 1). Collectively, although there has been no study comparing its efficacy with an anticancer drug, *O. japonica* has been shown to exert beneficial antiproliferative and cytotoxic properties on different cancer cell lines. With respect to its possible underlying mechanisms, the inhibitory effects of *O. japonica* extract on cancer cell proliferation and growth can be explained by pro-apoptotic induction and cell cycle blocking, because an imbalance between the two mechanisms leads to cancer development.

#### 3.1.1. Apoptosis Induction

Apoptosis, known as active programmed cell death, is characterized by morphological hallmarks, such as cell shrinkage, membrane blebbing, Ca^2+^ overload, chromatin condensation, DNA fragmentation, and apoptotic cell formation [24]. Cancer is a representative disease with relevance to the disabling of apoptotic responses [25]. Tumor cells proliferate by evading apoptotic signals and having resistance to apoptosis [25]. Therefore, apoptosis induction might be an essential mechanism of anticancer drugs to block excessive tumor cell proliferation by leading to selective cell death. Apoptosis can be induced via the activation of two caspase-dependent pathways: The death receptor-related extrinsic pathway and the mitochondria-mediated intrinsic pathway [26]. The intrinsic pathway involves the regulation of a variety of pro-apoptotic effectors (Bax, Bcl-xS, Hrk, Bak, Bid, Bik, and Bad) and anti-apoptotic factors (Bcl-2, Mcl-1, and Bcl-xL). Among these proteins, the interaction between Bax and Bcl-2 is crucial because it results in a loss of mitochondrial membrane potential, cytochrome c release, and caspase activation, eventually leading to apoptosis induction. Therefore, the matter of pro-apoptotic activities against cancer cells deserves considerable attention, especially in the development of novel anticancer drugs.

Mitogen-activated protein kinase (MAPK) signaling from outside the cell to inside the nucleus plays a crucial role in regulating cell proliferation, survival, and apoptosis. In particular, MAPK components (extracellular signal-regulated kinase (ERK)1/2, c-jun N-terminal kinase (JNK), and p38) mediate upstream cascades of mitochondria-mediated apoptosis, and MAPK activation can induce pro-apoptotic processes and/or anti-apoptosis [27]. In general, the phosphorylation of JNK and p38, which are more reactive to stress and cellular damage, facilitates the pro-apoptotic system, and ERK activation is mainly implicated in anti-apoptotic mechanisms. Therefore, JNK and p38 activation and ERK1/2 inhibition usually lead to cancer cell death [28]. However, the opposite has also been frequently reported [27].

In addition to regulating MAPK, p53 activation has also been reported to sensitize tumor cells to apoptosis, and it may target both extrinsic and intrinsic apoptotic factors. Indeed, p53 seems to modulate apoptotic regulatory proteins such as Bcl-xL, Bcl-2, and Bax [29]. p53 also enhances the expression of Bid, which leads to the convergence of both pathways [30]. In particular, the p53-induced translocation of Bid to mitochondria can improve the drug resistance of cancer cells against chemotherapeutic treatment [30].

*O. japonica* has been reported to block tumor proliferation in different cancer cells by efficiently restoring deregulated apoptosis and removing damaged cells (Table 1).

First, morphological analysis using fluorescence microscopy or Hoechst staining revealed that *O. japonica* treatment induced a series of distinctive morphological traits that were displayed in apoptotic cells in certain tumor cells, which are the main consequences of pro-apoptosis. In particular, both early phase (chromatin condensation and nuclear fragmentation) and late-stage (apoptotic body formation) morphological changes of the apoptotic trigger [31] were observed in the presence of *O. japonica* extract in AGS [17], HepG2 [32], SW480 [33], RC-58T/h/SA#4 [34], and PANC-1 [22] cancer cell lines.

Second, the antiproliferative roles of *O. japonica* in inhibiting cancerous cell growth were closely related to Bax and Bcl-2 expression; as such, *O. japonica* might be an antitumor agent targeting caspases. Subsequent Bax activation and Bcl-2 suppression induced by *O. japonica* allowed mitochondrial depolarization and cytochrome c release into the cytoplasm, which induced caspase-3 activation through mitochondria-mediated intrinsic apoptosis in AGS [17,35], HepG2 [32], SNU-1079 [36], A549 [37], HT-29 [38], SW480 [33], HeLa [39], OVCAR-3 [40], MDA-MB-231 [41], THP-1 [42], U937 [43], and K562 [44] cells. Interestingly, the treatment of AGS [35], HepG2 [32], SNU-1079 [36], HT-29 [38], HeLa [39], PANC-1 [22], and U937 [43] cells with *O. japonica* extract eventually enhanced the activity of caspase-8 by executing extrinsic apoptosis, as well as caspase-9, initiating the intrinsic pathway. Additionally, *O. japonica* extract enhanced tBid mitochondrial accumulation in SW480 cells, thereby suggesting its potential to converge both pathways.

Third, an upstream MAPK pathway was involved in the molecular mechanism of *O. japonica*-induced apoptosis in various cancer cells. The phosphorylation of p-38, p-JNK, and p-ERK was observed in HT-29 colon and PANC-1 pancreatic cancer cells after treatment with the ethyl acetate fraction of the 95% ethanol extract of *O. japonica* [22,38]. Its extract increased the level of p-p38 MAPK protein more markedly in gynecologic cancer and leukemia, such as in HeLa, OVCAR-3, MDA-MB-231, THP-1, and U937 cells. The decreased level of p-ERK1/2 protein induced by *O. japonica* in OVCAR-3 cells was inversely elevated in HepG2, HT-29, and MDA-MB-231 cells. *O. japonica* might therefore contribute to the pro-apoptotic or anti-apoptotic role of ERK, depending on the cancer cell lines. Moreover, *O. japonica* treatment enhanced the cellular abundance of the tumor suppressor protein p53 in AGS, A549, OVCAR-3, HL60, and L1210 cells. Therefore, the induction of apoptosis by *O. japonica* to inhibit cancer cells might be partly attributed to the MAPK pathway, which increases p53 expression and p53-dependent apoptosis.

Additionally, *O. japonica* inhibited tumor-specific metabolism in proliferating cancer cells by suppressing the expression of α-enolase, phosphoglycerate dehydrogenase (a regulator of the serine synthesis pathway), and fatty acid synthase, resulting in lower biosynthesis demands from cancers. The ability for *O. japonica* to reverse abnormal metabolism can contribute to the induction of apoptosis and selective toxicity in tumor cells.

**Figure 1:**
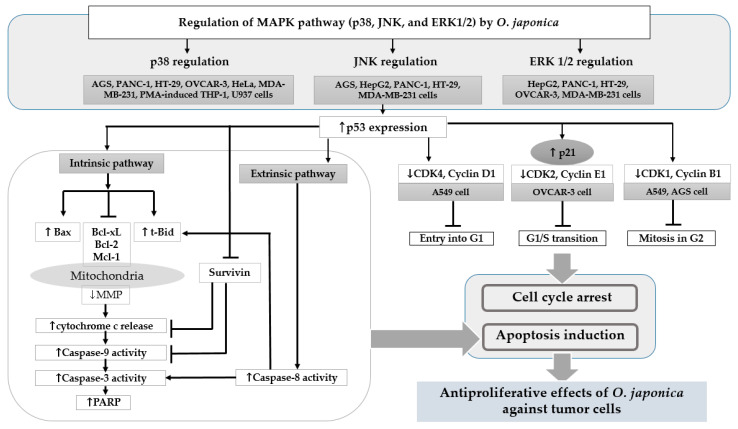
Mechanism of pro-apoptosis and cell cycle arrest induced by *Orostachys japonica* related to its antiproliferative effects. As shown, the regulation of MAPK expression in the presence of *O. japonica* against tumor cells causes an increase in p53 tumor suppressor gene expression. This modulation also results in apoptosis induction through intrinsic/extrinsic pathways and serine, fatty acid, and glucose metabolism in tumors. Similarly, cell cycle arrest at G1, the G1/S transition, and the G2 phase is induced by decreasing the expression of cyclins and CDKs. MAPK: mitogen-activated protein kinases; Bax: Bcl2-associated X protein; Bcl: B-cell lymphoma; Mcl: myeloid cell leukemia; t-Bid: truncated BH3-interacting domain death agonist; MMP: matrix metallopeptidases; PARP: poly ADP ribose polymerase; PHGDH: D-3-phosphoglycerate dehydrogenase; FASN: fatty acid synthase; CDK: cyclin-dependent kinase.

Consequently, the antiproliferative effects of *O. japonica* against cancer cells strongly depend on its potency to trigger apoptosis in target cells (Table 1). A simplified overview of the pro-apoptotic activities induced by *O. japonica* is depicted in Figure 1, summarizing the molecular mechanism of *O. japonica*-induced apoptosis. Eventually, *O. japonica* might modulate the MAPK signaling pathway to trigger the apoptosis machinery in cancer cells in response to lethal stimuli, such as DNA damage. It may also alter the resistance of incessantly proliferating tumor cells to apoptosis via a p53-dependent pathway involving caspase activation.

#### 3.1.2. Cell Cycle Arrest

The cell division cycle commonly proceeds from the G0/G1, S, G2, and M phases through the periodic expression of cyclin-dependent kinases (CDKs) and their partner cyclins. Cell cycle arrest can be initiated to repair impaired DNA damage, and the cell cycle resumes once recovery is completed [45]. However, unlike normal cells, cancer cells are characterized by excessive proliferation induced by the deregulation of cell cycle checkpoint control and rapid cell division without a repair process [46]. In particular, cell cycle aberrations in neoplastic cells are closely associated with the emergence of resistance to anticancer agents and tumor recurrence [47].

New strategies targeting the cell cycle-related CDKs and cyclin in cancer have received growing interest, because a series of CDKs or cyclin may be a direct cause of the tumorigenesis of normal cells into tumor cells [19]. Pan-CDK inhibitors (flavopiridol, R-roscovitine, P276-00, and SNS-032) and selective CDK inhibitors (palbociclib, dinaciclib, ribociclib, and abemaciclib) are under development, but these CDK drugs have been reported to exhibit a low specificity or cause adverse effects, such as leukopenia, neutropenia, and lung inflammation [48,49]

The antiproliferative effects of *O. japonica* against several cancer cells might be partly due to its ability to regulate the inappropriate overexpression of some CDKs and cyclins (Table 1). Firstly, the ethyl acetate fraction of the 95% ethanol extract of *O. japonica* selectively inhibited cyclin D1 and CDK4 expression, blocking entry into the G1 phase in A549, MDA-MB-231, A375, and PANC-1 cancer cells. Cyclin D1 plays a critical role as an oncogene; in particular, an increase in CDK4-cyclin D1 complex levels is an indicator of mammary carcinogenesis [50]. In this regard, it is possible to speculate that *O. japonica* might have preventive potential and offer better therapeutic effects against breast cancer, such as MDA-MB-231, via the suppression of protein expression in the early cell cycle.

Similar bioactivity of *O. japonica* targeting cell cycle regulators was found in OVCAR-3 and AGS cancer cells. In OVCAR-3 cells, *O. japonica* disturbed the G1/S transition and DNA synthesis in the S phase by downregulating CDK2 and cyclin E1 overexpression. In addition, *O. japonica* interfered with the progression of the later phase (M phase) by suppressing CDK1 and cyclin B1 levels in A549, AGS, and PANC-1 cells. This activity prevented the splitting of cells into two identical daughter cells and inhibited continual cancer cell proliferation.

Interestingly, the molecular mechanism underlying the antiproliferative functions of *O. japonica* against A549, OVCAR-3, and AGS cancer cells was associated with altered p53 expression. The most prominent outcome of loss of p53 function in cancer is impairment of the cell cycle and apoptosis [51]. In AGS gastric cancer, *O. japonica* increased the protein levels of p-p38 and p-JNK MAPK and promoted p53 activity, the markers of which were suppressed; AGS gastric tumor growth was thereby enhanced [35]. The regulation of the p38 and JNK MAPK-p53 pathways by *O. japonica* in AGS gastric cancer cells resulted in the transcriptional activation of CDK1 and cyclin B1, which in turn inhibited the transition from G2 to mitotic M phase entry [35]. In addition, *O. japonica* increased the accumulation of p53 protein in A549 cells, leading to the dose-dependent inhibition of CDK4-cyclin D1 and CDK1-cyclin B1 complexes and consequent G1 and G2/M arrest [37]. As shown in Figure 1, the suppression of CDK and cyclin induced by *O. japonica* may be associated with the role of p21 and p16 in response to p53 activation [49]. In OVCAR-3 cells, the ethyl acetate fraction of the 95% ethanol extract of *O. japonica* enhanced the mRNA levels of the tumor suppressor p53 and CDK inhibitor p21 through the effective combination of p-p38 and p-extracellular-signal-regulated kinase (ERK)1/2 MAPK, resulting in cell arrest during the G1/S phase and thus inhibiting cancer cell proliferation.

Taken together, proper modulation of the cancer cell cycle machinery may be one of the crucial mechanisms demonstrating the antiproliferative effects of *O. japonica* against tumor cells. Effective cell cycle arrest induced by *O. japonica* implies that its extract could inhibit CDK/cyclin hyperactivation-induced cancer cell development, proliferation, and progression by allowing no time to repair DNA injury. Moreover, *O. japonica* can prevent drug resistance in combined therapy with anticancer agents, as the emergence of chemotherapy resistance could be accelerated by cell cycle arrest [47] (Table 1). Therefore, it is possible to assume that *O. japonica* can act as a p53-dependent CDK-inhibiting anticancer drug, and that its involvement in p53 activity can be a key mechanism simultaneously mediating the cell cycle and apoptosis to suppress cancer survival.

### 3.2. Anti-Angiogenic Effect

Physiological vasculogenesis is a vital and normal process for forming new blood vessels during embryo development, the female ovarian cycle, and wound healing [62]. However, abnormal angiogenesis in which cells proliferate from pre-existing vessels and not stem cells is closely associated with the development of diabetic retinopathy, rheumatoid arthritis, psoriasis, and chronic inflammation [63]. In particular, tumor-derived angiogenesis accelerates the rapid growth, malignancy, microvasculature, and metastatic spread of cancer cells by supplying additional oxygen and nutrients [64]. Because of the key roles of endothelial cells in the tumor microenvironment, anti-angiogenic therapy has been introduced as a new strategy to specifically attack only activated endothelial cells, without affecting normal cells. However, angiogenesis inhibitors such as bevacizumab (Avastin^®^), afilbercept (Eylea^®^), and sorafenib (Nexavar^®^) offer challenges to patients due to their high cost and unfavorable side effects; these side effects include bleeding, proteinuria, hypertension, and diarrhea [65]. Therefore, a novel and cost-effective anti-angiogenesis therapy with a low toxicity is needed.

Angiogenesis consists of complex and multifactorial processes. Activated endothelial cells for angiogenesis require a response to vascular endothelial growth factor (VEGF) secretion from tumor cells, the production of proteolytic enzymes such as matrix metalloproteinases (MMPs), migration, adhesion, and tube formation (molecular mediators of angiogenesis). First, the 70% ethanol extract of *O. japonica* suppressed the proliferation of VEGF-stimulated human umbilical vein endothelial cells (HUVECs) and angiogenic sprouting from the Sprague Dawley (SD) rat aortic rings without toxicity [11]. The inhibitory actions of *O. japonica* against angiogenesis were consistently observed in VEGF-induced C57BL/6 mice [66] and xenograft BALB/c mice [52] implanted with prostate cancer cells by decreasing the number of infiltrating vessels into the Matrigel plug. Next, *O. japonica* extract decreased the level of MMP-9 protein and elevated TIMP-1 protein expression in HUVECs, which might prevent endothelial cells from escaping from pre-existing capillaries and moving to tumor tissue [11]. In addition, the 70% methanol extract of *O. japonica* fermented with *Aspergillus kawachii* was excellent for lowering the number of migrated cells required for invasive cancer; this was observed using a wound healing assay and Transwell migration assay in Ms-1 endothelial cells via focal adhesion kinase (FAK) and Src inactivation [66]. Similarly, migrated cells were diminished in the presence of the 70% ethanol extract of *O. japonica* in VEGF-stimulated HUVECs through the blockade of FAK/Src, phosphoinositide 3-kinase (PI3K)/protein kinase B (Atk)/mammalian target of rapamycin (mTOR), and MAPK [11]. Furthermore, *O. japonica* improved key angiogenic processes, such as the differentiation of endothelial cells into the capillary web structure and tube formation, in Ms-1 cells and HUVECs [61,66] (Table 2).

The anti-angiogenic effects of *O. japonica* against cancer cells were involved in not only endothelial cell activation, but also angiogenic stimulation from tumor cells. In HepG2 cells, an *O. japonica* water extract reduced both the secretion of VEGF from HepG2 cells and the intracellular production of VEGF and basic fibroblast growth factor (bFGF) via the regulation of ERK and Akt [67]. In addition, its extract significantly altered VEGF and bFGF mRNA levels in HepG2 cells, even in a hypoxic chamber vulnerable to tumor progression [67]. The reduction of VEGF secretion from cancer cells by *O. japonica* can suppress the VEGF-VEGFR2 pathway in endothelial cells, thereby inhibiting angiogenesis initiation.

**Figure 2:**
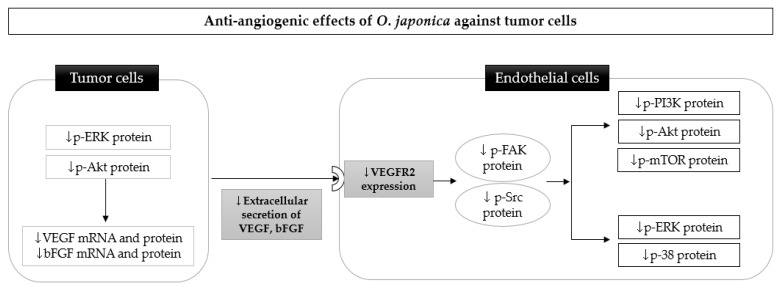
Possible molecular mechanism underlying the anti-angiogenic effects of *Orostachys japonica* against tumor cells, primarily, a blockade of the VEGF-VEGFR2 pathway. The downregulation of ERK and Akt by *O. japonica* in tumor cells results in the inhibition of VEGF and bFGF expression. The reduction of VEGF secretion from tumor cells by *O. japonica* can suppress the VEGF-VEGFR2 pathway in endothelial cells, thereby sequentially inhibiting phosphorylation of the FAK and Src, PI3K/Akt/mTOR, and MAPK pathways. ERK: extracellular signal-regulated kinases; Akt: protein kinase B; VEGF: vascular endothelial growth factor; bFGF: basic fibroblast growth factor; FAK: focal adhesion kinase; Src: proto-oncogene tyrosine-protein; PI3K: phosphoinositide 3-kinases; mTOR: mammalian target of rapamycin.

Overall, *O. japonica* could serve as a potential angiogenesis inhibitor that exhibits pleotropic effects targeting multiple processes of the proliferation, migration, invasion, and cell tube formation of endothelial cells, as well as the secretion of growth factors from tumor cells. The anti-angiogenic effects induced by *O. japonica* were chiefly mediated via the VEGFR2 signaling pathway. *O. japonica* reduced the stimulatory activity of VEGF on VEGFR2 in endothelial cells, sequentially leading to the inactivation of downstream pathways of VEGFR2, such as the FAK/Src, PI3K/Atk/mTOR, and MAPK pathways. Therefore, as the proliferation, migration, and invasiveness of endothelial cells in response to VEGFR2 overexpression can be potentially regulated by *O. japonica*, this extract is expected to play a role as a VEGFR2 inhibitor to develop specific and selective anticancer agents (Figure 2).

### 3.3. Antimetastatic Effect

Metastasis is a complex process involving the detachment of cancer cells from the primary sites, migration, and invasion into the secondary organs [68]. The metastatic cascade is responsible for more than 90% of cancer-related deaths [69], as very few of the more than 200 approved drugs induce tumor shrinkage, block angiogenesis, and enhance immune system control carcinoma metastasis [70]. Therefore, effectively suppressing the metastasis of different tumor cells is critical for increasing the survival rate of cancer patients, especially in terms of the early detection of tumors.

One of the main biological mechanisms responsible for the initiation and completion of cancer metastasis is the epithelial-to-mesenchymal transition (EMT) process by which cells lose epithelial junctions and acquire mesenchymal characteristics, facilitating their migration and invasion [71]. EMT has been shown to promote the dissemination of tumor cells at distant sites, and is known to be the leading cause of cancer-associated mortality. During EMT, the expression of extracellular matrix (ECM)-associated proteases such as MMPs, the tissue inhibitor of MMP (TIMP), and the urokinase-type plasminogen activator (uPA) are changed to degrade ECM stiffness, resulting in cell protrusion, migration, and invasion [72].

The most remarkable finding regarding the antimetastatic role of *O. japonica* in cancer cells is that its extract is effective in suppressing the overexpression of MMP-1, -2, and -9 in THP-1 leukemia [73], HT1080 human fibrosarcoma [74], and LnCaP prostate cancer cells [75]. In addition, a similar pattern of uPA protein expression and an opposite increase in TIMP-1/2 mRNA and protein levels were observed in RC-58T/h/SA#4 primary prostate cancer cells [52] and LnCaP prostate cancer cells [75], respectively, in the presence of *O. japonica*. These findings suggest that *O. japonica* might inhibit the pro-metastatic functions of cancer cells by recovering their ECM structure, thus preventing cancer metastasis. In particular, the inhibitory effects of *O. japonica* against cancer migration and invasion have been consistently observed via wound healing and Transwell invasion assays in highly invasive tumor cells, such as MDA-MB-231 breast cancer [41], SW480 colon cancer [58], HT1080 fibrosarcoma [74], LnCaP [75], and RC-58T/h/SA#4 prostate cancer [52] (Table 3).

*O. japonica* appears to block EMT and its related proteins via the FAK-stimulated PI3K/Akt/mTOR and MAPK pathways, which are closely involved in NF-κB activation in EMT and metastasis. Interestingly, *O. japonica* was shown to lower the number of migrating and invading cells in a hypoxic tumor microenvironment, accelerating tumor growth and metastasis by suppressing hypoxia-inducible factor (HIF)-1α signaling. This suppression was accompanied by the blockade of FAK-stimulated PI3K/Akt/mTOR and MAPK phosphorylation [52] (Table 3).

In addition to the bioactive intervention of *O. japonica* in EMT-related targets, another study demonstrated that its methanol extract suppressed platelet aggregation induced by adenosine diphosphate [76]; as platelets can activate cancer proliferation and metastasis, this effect inhibited tumor metastasis [77]. Moreover, an *O. japonica* extract hampered B16-Fo lung cancer cell adhesion to a single ECM such as laminin, suggesting that it can prevent the onset of cancer metastasis by decreasing adherent cells bound to the ECM [76] (Table 3).

In tumorigenesis, metastasis is important for cancer progression, and EMT plays a critical role in strengthening the metastatic ability of malignant cells. EMT-related MMPs, uPA, and TIMPs were regulated by *O. japonica* treatment, accompanied by a decrease in the number of migrated cells in various cancer cells. The pharmacological activities of *O. japonica* in response to EMT might be implicated in the inhibition of FAK-induced PI3K/Akt/mTOR and MAPK activation (Table 3). Overall, *O. japonica* can target EMT-driven markers and signaling pathways to suppress the metastasis formation of tumors, and could serve as an antimetastatic drug that inhibits the disruption of the structure of ECM at the primary site and prevents cancer cell dissemination. However, the activities of *O. japonica* against metastasis that do not require the EMT of tumor cells and are triggered by cancer cell clusters are unclear.

## 4. Possibility of the Combined Use of *O. japonica* with Other Chemotherapeutic Drugs

The effectiveness of currently available anticancer drugs does not meet expectations due to their non-specific cytotoxicity and low specificity [78]. However, whether using a traditional herbal formula as an adjuvant therapy to conventional drugs can potentiate the efficacy of chemotherapy and reduce adverse effects is controversial. A recent systematic review raised questions regarding the efficacy of herbal decoctions combined with conventional chemotherapeutic agents [79]. However, combinational therapeutic strategies to enhance anticancer properties and decrease side effects have received attention. In particular, many medicinal herbs, including *Astragulus* [80] and *Carthamus tinctorius* [81], are considered useful adjuvants for enhancing the cellular immune response and antitumor activities of cancer therapy. Among them, *O. japonica* could work complementarily and synergistically when used in combination with other anticancer drugs for a number of reasons.

First, *O. japonica* can improve the immune system in cancer pathogenesis. In immunosuppressed Wistar rats treated with cyclophosphamide, both the water and 70% ethanol extract of *O. japonica* elevated the decreased weights of the thymus and spleen, white blood cell counts in peripheral blood, and serum immune-related cytokines (TNF-α, IL-2, and IFN-γ), which are closely associated with cyclophosphamide-induced immunosuppression [82]. Herbal acupuncture with *O. japonica* increased Th1-related IFN-γ levels in splenocytes and elevated IL-12 and IFN-γ production in Balb/c mice injected with Colon26-L5 cancer cells [57]. These results suggest that *O. japonica* might stimulate the activation of immune cells to elicit an enhanced immune response, which may have been suppressed during chemotherapeutic regimens against cancer. In addition, the immunostimulatory effects of *O. japonica* can potentiate the antiproliferative effects of anticancer drugs when used in combined therapy, as tumor cells develop and proliferate by avoiding the action of immune cells.

Second, *O. japonica* can be administered in combination with anticancer drugs due to its anti-inflammatory effects. Inflammatory pathogenesis induced by chemotherapy drugs such as doxorubicin, cisplatin, and 5-fluorouracil might be the leading cause of cancer metastasis and treatment failure [83]. Although it is not a chemotherapy-enhanced inflammation experimental model, *O. japonica* reversed tumor-related perturbations of cytokines. The 80% methanol extract inhibited the expression of proinflammatory factors in LPS-induced THP-1 leukemia cells via NF-κB and MAPK suppression [84]. Similarly, the ethyl acetate and *n*-butanol extract of *O. japonica* downregulated IL-8 and COX-2 production by blocking the NF-κB and MAPK pathways in TNF-stimulated HT-29 colon cancer cells [85].

Therefore, combining *O. japonica* with conventional chemotherapeutic agents to treat cancer may be useful; its extract has been found to possess immunostimulatory and anti-inflammatory bioactivity, which is helpful in combined cancer management. From existing laboratory evidence, further investigations focusing on its efficacy, the contribution of MAPK, safety, and quality control are required to broaden its clinical application in cancer management.

## 5. Anticancer Effects of Two Representative Flavonoids from *O. japonica*

**Figure 3:**
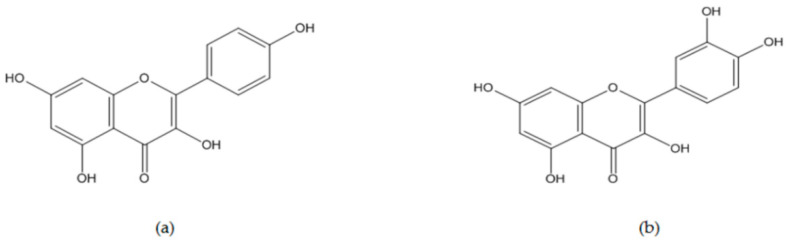
Chemical structures of kaempferol and quercetin belonging to flavonoids from *Orostachys japonica*: (**a**) Kaempferol (3,5,7-trihydroxy-2-(4-hydroxyphenyl)-4H-1-benzopyran-4-one, C_15_H_10_O_6_, molecular mass 286.23 g/mol), and (**b**) quercetin (3,3′,4′,5,7-pentahydroxyflavone C_15_H_10_O_7_, molecular mass 302.236 g/mol).

Sterols, flavonoids, aromatic acids, and triterpenoids have been isolated from *O. japonica*. Flavonoids are well-known polyphenols possessing strong antioxidant activities. On the other hand, compounds have been reported to stimulate oxidative stress by depending on copper ions in tumor cells, thus inducing nuclear DNA damage and tumor suppression [86]. Therefore, kaempferol and quercetin, which are major flavonoids obtained from *O. japonica*, may play a crucial role in its antitumor properties (Figure 3).

### 5.1. Kaempferol

Kaempferol (3,5,7-trihydroxy-2-(4-hydroxyphenyl)-4H-1-benzopyran-4-one, C_15_H_10_O_6_, molecular weight 286.23 g/mol, Figure 3) is a natural flavonol and phytoestrogen belonging to the flavonoids group, and it is abundantly found in fruits and vegetables. Kaempferol and its derivatives have been isolated from medicinal plants such as *Foeniculum vulgare* [87], *Castanea mollissima* Blume [88], and *O. japonica* [89], and it has been revealed to exert antioxidant, anti-inflammatory, antifungal, antidiabetic, cardioprotective, reno-protective, and anticancer effects [90]. In particular, it is well-established in the literature that kaempferol mainly triggers anticancer responses through modulation of the MAPK and Akt pathways, the activation of tumor suppressor genes such as PTEN and p53, and the downregulation of E-cadherin and vimentin, which are relevant to EMT in various carcinomas (breast cancer, cervical cancer, endometrial cancer, ovarian cancer, hepatocellular carcinoma, bile duct cancer, gastric cancer, colon cancer, pancreatic cancer, esophageal cancer, lung cancer, leukemia, bladder cancer, and osteosarcoma cells) [91,92,93].

Kaempferol has been revealed to enhance the efficacy of conventional chemotherapeutic drugs, and it reduces side effects and drug resistance when used in combination with kaempferol compared to single-drug treatment. Regarding the enhancement of its efficacy, kaempferol was found to potentiate doxorubicin-induced apoptosis in U87MG glioblastoma cells [94]. In addition, the antiproliferative effect of 5-fluorouracil was stronger when treated in combination with 100 and 35 µM of kaempferol in colon cancer (HCT-8 and HCT-116) and pancreatic cancer (MIA PaCa-2), respectively [95,96]. A similar synergistic effect of kaempferol (35 and 20 µM) was observed in ovarian cancer cells (A2780 and OVCAR-3) in the presence of cisplatin [97,98]. Moreover, the addition of kaempferol (75 and 2.5 µM) improved the drug resistance to 5-fluorouracil and sorafenib in colon cancer (5-FU-resistant LS174-R) and hepatocellular carcinoma (HepG2 and N1S1), respectively [99,100]. The development of adverse effects, such as doxorubicin-induced cardiotoxicity, endotheliotoxicity, cisplatin-induced nephrotoxicity, and hearing loss, was shown to be inhibited by co-treatment with kaempferol [101,102,103,104,105].

Taken together, kaempferol is an active compound exhibiting excellent antitumor activities via its complex roles in various stages of cancer, from cancer proliferation to progression and metastasis, and has potential for use in both single and complementary therapy.

### 5.2. Quercetin

Quercetin (3,3′,4′,5,7-pentahydroxyflavone C15H10O7, molecular weight 302.236 g/mol, Figure 3) is abundantly distributed in fruits and vegetables, and it is also categorized as a natural flavonol that contains kaempferol. The chemical structures of quercetin and kaempferol have a 3-hydroxy flavone backbone; however, quercetin has an additional hydroxyl group, thereby increasing its chemical reactivity [106]. Quercetin has been known to show antidiabetic [107], anti-Alzheimer [108], anti-inflammatory [109,110,111], and antiviral activities [112]. Notably, it was reported that a low concentration of quercetin acted as an antioxidant, but high-dose quercetin induced oxidative stress against cancer cells, thus displaying anticancer effects [113]. Recent reports have shown that quercetin is excellent in combating ovarian cancer [114] and hepatocarcinoma [115]. In addition, quercetin inhibited the overgrowth of human gastric cancer cells (in the presence of 53 µM quercetin) [116], MDA-MB-453 (in the presence of 100 µM quercetin) [117], and MCF-7 (in the presence of 150 µM quercetin) [118] via the induction of intrinsic and extrinsic apoptosis pathways and cell cycle arrest. Moreover, cancer-related alterations of angiogenic and metastatic mediators were reversed by quercetin in prostate cancers [119,120].

Accumulating evidence regarding the synergistic effects of quercetin in combined therapy has revealed that the compound could enhance the antiproliferative effects of doxorubicin [121,122,123], cisplatin [124], 5-fluorouracil [125], and sorafenib [126] against various cancers. In particular, quercetin inhibited the growth of cancer stem cells and angiogenesis more potently than treatment with doxorubin [121,123] and SN-38 [127] alone. Considering the occurrence of drug resistance in cancers exposed to chemotherapeutic agents, quercetin reversed multidrug resistance during doxorubicin treatment [128] and suppressed cisplatin-induced CYP1B1 overexpression [129]. Quercetin was also beneficial in alleviating testicular and cardiac toxicity induced by doxorubicin [130,131] and cyclophosphamide teratogenesis [132].

Conclusively, these results show that quercetin might serve as a useful anticancer drug and chemosensitizer by increasing p53 expression, targeting VEGFR2 signaling pathways, and inhibiting the PI3K and Wnt pathways.

## 6. Safety

As most anticancer drugs are expected to induce considerable toxicity to normal cells as well as cancer cells, it is important for anticancer chemotherapeutic agents to have safe pharmacology. In the case of anticancer drugs targeting tumor cell proliferation, adverse reactions, such as nausea, vomiting, and hair loss, have been commonly observed as a result of inevitable attacks on fast-growing epithelial cells in hair-follicle and gastrointestinal surfaces [133]. As mentioned above, *O. japonica* has been reported to exhibit various pharmacological effects that could improve tumor-related pathogenesis, resulting in tumor development and progression. Interestingly, an aqueous *O. japonica* extract (250 mg/kg, orally administered for 12 weeks) exhibited no significant toxicity-related symptoms or results in SD rats [134]. In an acute oral toxicity test of its ethyl acetate fraction (500, 1000, and 2000 mg/kg, orally administered for 14 days) in Balb/c mice, no significant changes in serum markers and histological findings were observed. Its 50% lethal dose (LD50) in the study was above 2000 mg/kg [135], which suggests that its extract can be administered without harmful toxicity up to a high dose for short-term use.

In particular, it is noteworthy that the 95% methanol extract of *O. japonica* did not show any cellular toxicity in 293 kidney epithelial cells up to 100 μg/mL, but cell proliferation of HL60 human acute promyelocytic leukemia was significantly inhibited in a dose-dependent manner in the presence of 10 and 100 μg/mL of its extract [54]. This suggests that *O. japonica* can act differently, depending on the cell types.

In summary, based on in vivo studies using a 250–2000 mg/kg dose of *O. japonica*, the toxic impact of its supplement might be insignificant. Additionally, *O. japonica* was introduced as a safe medicinal plant that can be used as food in the database for raw food materials from the Korea Food and Drug Administration [136]. Furthermore, *O. japonica* may exhibit specific responses to the type of cancer cells, and may not be toxic to normal cells. Therefore, further in-depth studies are required to clarify its safety and determine the optimal doses of *O. japonica* and its bioactive compounds for its broad application in managing cancer patients.

## 7. Discussion

In the current review, we conducted a literature review to discern the antitumor effects and underlying cellular mechanisms of *O. japonica* and two of its compounds. This approach was based on available experimental studies reporting significant outcomes in the presence of these materials in tumor cells. The use of *O. japonica* has been found to exhibit pharmacological activities in preclinical cancer studies, with selective toxicity towards malignant cells compared with normal cells. Interestingly, *O. japonica* not only inhibited an unconstrained growth of tumors through apoptosis, cell cycle arrest, and targeting metabolism, but also affected cancer-related angiogenesis and metastasis.

In particular, the MAPK signaling pathways play a crucial role in the physiology and pathogenesis of various cancers, including tumor proliferation, angiogenesis, and EMT-driven metastasis. MAPK signaling is often deregulated in tumors by responding to a wide array of molecular changes [26]. Regarding the fundamental proliferative feature of tumors, upregulated ERK of MAPK in cancer mainly plays a pivotal role in accelerating cell proliferation [137]. Altered MAPK signaling is involved in the hindrance of apoptosis and cell cycle arrest, which are classic mechanisms contributing to tumor proliferation [138]. Moreover, VEGF expression important in uncontrolled angiogenesis in tumors frequently depends on MAPK [139] and p38γ MAPK is known to enhance EMT and augment cancer metastasis and aggressiveness [140]. Therefore, targeting MAPK signaling might be efficient for managing cancer. The interaction of *O. japonica* with MAPK components facilitates the pro-apoptotic changes and cell cycle arrest of various cancer cells, which in turn results in tumor cell death. In addition, alterations of MAPK were subsequently induced by a blockade of the VEGF-VEGFR2 pathways in the presence of *O. japonica* extract. Moreover, the biological actions of *O. japonica* targeting EMT-stimulated metastasis are closely involved in FAK-induced PI3K/Akt/mTOR and MAPK activation.

Similarly, kaempferol and quercetin have been shown to have a strong capacity to control tumorigenesis and progression via the regulation of tumor suppressor genes and molecular pathways, such as NF-κB, MAPK, and PI3K/Akt/mTOR. Therefore, the major active compounds contributing to the anticancer potential of *O. japonica* may be kaempferol and quercetin. However, in some studies, a low bioavailability of these two flavonols has been reported due to poor oral absorption [106]. Therefore, the impact of extraction methods and solvents used to obtain *O. japonica* extract on the bioavailability of kaempferol, quercetin, and other compounds should be considered for further investigation.

As mentioned above, the concomitant use of *O. japonica* can be a more effective therapeutic strategy compared with treatment with conventional chemotherapeutic drugs alone. The recovery of cyclophosphamide-induced immunosuppression was observed in combination with both water and a 70% ethanol extract of *O. japonica*. Kaempferol and quercetin enhanced the sensitivity of cancer cells towards chemotherapeutic agents and ameliorated adverse effects, with an enhanced efficacy upon combined use with conventional anticancer regimens. Therefore, *O. japonica* might play a role as a promising adjuvant for chemotherapeutic applications, as well as an independent therapy.

**Figure 4:**
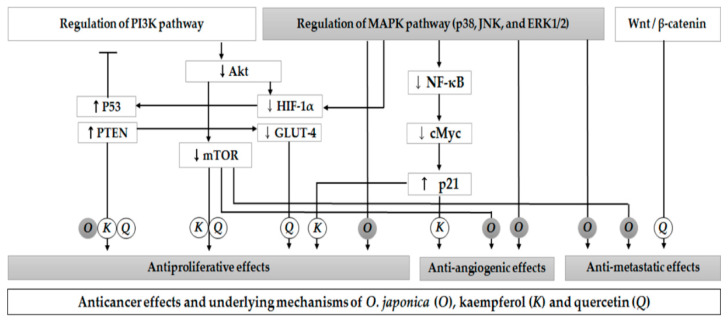
Anticancer effects and underlying molecular mechanisms of *Orostachys japonica* (O) and its two compounds (kaempferol (K) and quercetin (Q)). *O. japonica* increases the expression of tumor suppression gene p53 and regulates MAPK pathways, which involves the PI3K pathway and cancer glycolysis. These pathways may therefore control the expression of various markers contributing to the anticancer effects of *O. japonica*. Kaempferol and quercetin are shown to exhibit antiproliferative effects via PI3K signaling cascades. Quercetin inhibited HIF-1α and GLUT-4 expression and regulated Wnt/β-catenin. Kaempferol inhibited NF-κB and c-Myc expression and upregulated p21 activity, thus suppressing tumor growth and angiogenesis. PTEN: phosphatase and tensin homolog; HIF: hypoxia-inducible factors; GLUT: glucose transporter; NF-κB: nuclear factor kappa-light-chain-enhancer of activated B cells; c-Myc: cellular Myc.

In summary, we reviewed the antitumor activities and molecular cascades of *O. japonica* and its two compounds in tumor-related cellular and animal models. These compounds showed multitargeted efficacy influencing various stages, such as tumor cell proliferation, angiogenesis, and metastasis. The antitumor mechanisms of *O. japonica* and its two compounds are illustrated in Figure 4, which can be attributed to the reversal of the expression of indicators related to cancer cell toxicity, endothelial cell activation, and EMT-related mediators by regulating the downstream cascades of MAPK (p38, JNK, and ERK1/2). In particular, elevated levels of PTEN and p53 and the regulation of PI3K/Akt/mTOR molecules might constitute important signaling downstream critical for the efficacy and mechanism of kaempferol and quercetin potential in attacking tumors, thus being implicated in the MAPK pathway. Despite the antitumor efficacy of the two compounds, their clinical usage might be limited due to a low bioavailability. Instead, the use of *O. japonica* might be more efficient than a single-ingredient intake. A recent study demonstrated that a subcritical water extract of *O. japonica* at the condition of 220 °C for 15 min was revealed to contain the highest contents of phenolics and flavonoids, which are excellent for showing antioxidant activities [89]. Its extract can be administered to patients because taking boiled herbs with water is common in clinical settings. In addition, *O. japonica*-based combination therapies may be more efficient as a promising adjuvant to standard therapy. Our reviews also showed that *O. japonica* is considered to be selectively toxic to cancer, so it has fewer adverse effects than existing anticancer drugs. However, because recent studies investigating the safety of *O. japonica* have some limitations due to low dosages, short investigation periods, and insufficient outcomes, more elaborate safety evaluation is required.

## 8. Conclusions

Supported by a large amount of preclinical evidence related to the pharmacological effects of *O. japonica* against cancer, the present review has demonstrated that *O. japonica* and its two compounds—kaempferol and quercetin—exert potent antitumor effects against cancer. The anticancer mechanisms of *O. japonica* include apoptosis, cell cycle arrest, a blockade of VEGFR2 signaling, and EMT targeting, all of which are associated with the modulation of MAPK pathways. In addition, kaempferol and quercetin derived from *O. japonica* extract, which have been extensively found to show strong antitumor effects, might control different types of cancers via a couple of mechanisms including MAPK components. For the clinical use of these compounds and *O. japonica* extracts, setting standards by which extraction and formulation will be performed is required for ensuring a higher efficacy. Furthermore, based on increasing preclinical outcomes in the presence of *O. japonica* against a tumor and its standardization data, well-designed clinical trials should be conducted to evaluate the anticancer effects and provide a high level of evidence for its pharmacological application.

## Figures and Tables

**Table 1:** Antiproliferative effects and molecular mechanisms of *O. japonica.*

Extract	Model	Concentration	Result	Mechanism	Ref
E,W	SNU-1079 (in vitro)	300 μg/mL	↓Intrahepatic cholangio-carcinoma cells	↑Early and late apoptosis rate ↓Bcl-2, Mcl-1, Survivin mRNA ↑Bax mRNA ↑Cleaved caspase -3	[36]
				Cell cycle arrest ↓Cyclin D1 mRNA	
E	PMA-induced THP-1 cells (in vitro)	300 μg/mL	↓Leukemia cells	↑Early and late apoptosis rate ↓Bcl-2, Mcl-1, Survivin mRNA ↑Bax mRNA ↑Cleaved caspase-3	[42]
				Autophagy ↑LC3 II, beclin-1 ↓Atg5	
				MAPK pathway ↑p38 protein	
				↓NF-κB ↓mTOR mRNA	
EA	AGS (in vitro)	100, 200 μg/mL	↓Gastric cancer cells (IC_50_: 86 μg/mL)	↑Early and late apoptosis rate ↑DNA fragmentation ↑Apoptotic bodies ↓Bcl-2 ↑Cleaved caspase-3	[17]
				Cell cycle arrest Sub-G1 peak and G2/M arrest	
				↑Tumor suppressor p53	
EA	AGS (in vitro)	100 μg/mL	↓Gastric cancer cells	↑Early and late apoptosis rate ↓Bcl-2 ↑Cytochrome c ↓Pro-caspase-3,8,9 ↑Cleaved caspase-3,8,9	[35]
				Cell cycle arrest Sub-G1 peak and G2/M arrest ↓Cyclin B1, CDK1 mRNA	
				MAPK pathway ↑p38, JNK protein	
				↑Tumor suppressor p53	
EA	HeLa (in vitro)	10 μg/mL	↓Cervical cancer cells	↑Early and late apoptosis rate ↓Bcl-2 ↑Cytochrome c ↓Pro-caspase-3,8,9 ↑Cleaved caspase-3,8,9	[39]
				Cell cycle arrest Sub-G1 peak and G2/M arrest ↓Cyclin B1, CDK1 mRNA	
				MAPK pathway ↑p38, JNK protein	
EA	HepG2 (in vitro)	100, 200 μg/mL	↓Liver cancer cellsc	↑Early and late apoptosis rate ↑Condensed chromatin ↑Fragmented nuclei ↑Apoptotic bodies ↓Bcl-2 (not Bax) ↑Cytochrome c ↓Pro-caspase-3,8,9	[32]
				MAPK pathway ↑p-JNK, p-ERK1/2 protein	
EA	A549 (in vitro)	75, 100 μg/mL	↓Lung cancer cells	↑Early and late apoptosis rate ↑Condensed chromatin ↑Fragmented nuclei ↑Apoptotic bodies ↑Bax ↓Bcl-2	[37]
				Cell cycle arrest ↓Cyclin B1, CDK1, Cyclin D, CDK4 mRNA	
				↑Tumor suppressor p53 protein	
EA	HT-29 (in vitro)	50, 75, 100 μg/mL	↓Colon cancer cells	↑Early and late apoptosis rate ↑Apoptotic bodies ↑Bax ↓Bcl-2 ↑Cleaved caspase -3,8,9	[38]
				Cell cycle arrest Sub-G1 peak	
				MAPK pathway ↑p38, JNK, ERK1/2 protein	
EA	OVCAR-3 (in vitro)	50 μg/mL	↓Ovarian cancer cells	↑Early and late apoptosis rate ↑Apoptotic bodies ↑Bax/Bcl-2 ratio	[40]
				Cell cycle arrest Sub-G1 peak ↑p21 ↓Cyclin E1/CDK2 mRNA	
				MAPK pathway ↑p38, ERK1/2 protein	
				↑Tumor suppressor p53 protein	
EA	PANC-1 (in vitro)	50, 100 μg/mL	↓Pancreatic cancer cells (IC_50_: 50 μg/mL)	↑Early and late apoptosis rate ↑Condensed chromatin ↑Fragmented nuclei ↑Cytochrome c ↑Cleaved caspase-3,9 ↓Pro-caspase-3,8,9	[22]
				Cell cycle arrest Sub-G1 peak and G2/M arrest ↓Cyclin D1, Cyclin B1, CDK4	
				MAPK pathway ↑p38, JNK, ERK protein	
EA	MDA-MB-231 (in vitro)	20, 40, 60 μg/mL	↓Breast cancer cells	↑Early and late apoptosis rate ↑Condensed chromatin ↑Fragmented nuclei ↑Cytochrome c ↓Pro-caspase-3,8,9 ↑Cleaved caspase-3,9	[41]
				Cell cycle arrest Sub-G1 peak and G2/M arrest ↓Cyclin D1, Cyclin B1, CDK4	
				MAPK pathway ↑p38, JNK, ERK protein	
EA	A375 (in vitro)	120, 140, 150 μg/mL	↓Melanoma cells	Cell cycle arrest Sub-G1 peak ↓CDK1, cyclin B1 ↓CDK4, cyclin D	[23]
E	Male Balb/c mice xenografted RC-58T/h/SA#4 (in vivo)	25, 50 mg/kg	↓Prostate cancer size and volume	N.A.	[52]
M	L1210, U937 (in vitro)	100 μg/mL	↓Leukemia cells	↑Tumor suppressor p53 mRNA	[53]
				Cell cycle arrest Sub-G1 peak	
	Male Balb/c mice transplanted L1210 cells (in vivo)	500 mg/kg	↑Apoptosis rate of leukemia cells from peritoneal	↑Early and late apoptosis rate	
M	HL-60 (in vitro)	100 μg/mL	↓Leukemia cells	↑Early and late apoptosis rate	[54]
				Cell cycle arrest Sub-G1 peak	
				↑Tumor suppressor p53 gene/protein	
				↑NF-κB p50	
M	AGS, MCF-7 (in vitro)	300 μg/mL	↓Cancer cells	↑Early and late apoptosis rate ↑Mitochondria depolarization(ΔΨm) ↑Cleaved caspase-3	[55]
				Cell cycle arrest Sub-G1 peak	
M	SW480 (in vitro)	500 μg/mL	↓Colon cancer cells	↑Apoptosis ↑Apoptotic bodies ↑Cleaved caspase-3,9 ↓Bid ↑t-Bid ↑Bax ↓Bcl-2 ↑PARP	[33]
				Cell cycle arrest Sub-G1 peak	
M	RC-58T/h/SA#4 (in vitro)	300, 600 μg/mL	↓Prostate cancer cells ↑Floating cells	↑Early and late apoptosis rate ↑Condensed chromatin ↑Fragmented nuclei ↑Apoptotic bodies	[34]
				Cell cycle arrest Sub-G1 peak	
M	U937 (in vitro)	200, 400 μg/mL	↓Leukemia cells	↑Apoptosis ↓Pro-caspase-3,8,9 ↓MMP(ΔΨm), Bcl-2 ↓Bid, XIAP ↑PARP	[43]
				Cell cycle arrest Sub-G1 peak	
				MAPK pathway ↑p38 protein	
				↓p-Akt protein	
W	HepG2, Hep2B, AGS, KATOIII (in vitro)	20 μg/mL	↓Cancer cells ↑Floating cells	N.A.	[56]
W	Colon 26-L5 (in vitro)	300 μg/mL	↓Colon cancer cells	N.A.	[57]
W	SW480 (in vitro)	N.A.	↓Colon cancer cells ↑Floating cells	N.A.	[58]
	Male C57BL/6 mice transplanted SW480 cells (in vivo)	N.A.	↓Tumor weight and volume ↓Tumor formation	N.A.	
W	OVCAR-3, HeLa (in vitro)	100, 1000 μg/mL	↓Cancer cells	↑Apoptosis	[59]
				↓Cancer metabolism ↓hnRNP A2/B1 ↓Alpha-enolase ↓PHGDH	
				MAPK pathway ↑p38 protein	
W	K562 (in vitro)	100, μg/mL	↓Chronic myeloid leukemia cells	↑Early and late apoptosis rate ↑DNA fragmentation ↓Bcl-2 mRNA ↑Caspase-3 mRNA	[44]
W	HT-29 (in vitro)	2 mg/mL	↓Colon cancer cells shrunken, disintegrated, rounded, detached cells	↑Early and late apoptosis rate ↑Apoptosis gene (BAD, FADD, caspase-3,8,9) ↑Tumor suppression gene (TP53BP2 and STAT1) ↓Cell proliferation and growth gene (PTK6) ↓Anti-apoptosis gene (Bcl-2) ↓Cell cycle regulation gene (RFC5) ↓Cancer development gene (CTSH)	[60]
				Cell cycle arrest Sub-G1 peak and G2/M arrest	
W	A549, HeLa, AGS (in vitro)	0.5, 1 mg/mL	↓Cancer cells	↑Early and late apoptosis rate ↑DNA fragmentation	[61]

E: ethanol extract; M: methanol extract; W: water extract; EA; ethyl acetate fraction; MAPK: mitogen-activated protein kinases; Bax: Bcl2-associated X protein; Bcl: B-cell lymphoma; Mcl: myeloid cell leukemia; Akt: protein kinase B; ERK: extracellular-signal-regulated kinase. “↓” and “↑” represent the decrease or increase of the value.

**Table 2:** Anti-angiogenic effects and molecular mechanisms of *O. japonica.*

Extract	Inducer	Model	Concentration	Result	Mechanism	Ref
M	None	Ms-1 (in vitro)	100 μg/mL	↓Wound closure area ↓Migrated cells ↓Infiltrated capillary structure ↓Adherent cells	↓p-FAK protein ↓p- Src protein	[66]
M	VEGF 200 ng/mL	Male C57BL/6 mice (in vivo)	0.1 mg/mL	↓Blood vessels infiltrated into Matrigel plug	N.A.	
E, M, W, EA	VEGF 20 μg/mL	HUVECs (in vitro)	20 μg/mL	↓Wound closure area ↓Migrated cells ↓Infiltrated capillary structure	N.A.	[61]
E	VEGF 20 μg/mL	HUVECs (in vitro)	10~20 μg/mL	↓Wound closure area	↓p-VEGFR2 protein ↓p-FAK protein ↓p-Src protein ↓MMP-1, -9 protein ↓p-PI3K/p-Akt/p-mTOR protein ↓p-p38, p-ERK1/2 protein	[11]
E	VEGF-A 50 ng/mL	HUVECs (in vitro)	10~20 μg/mL	↓Migrated cells ↓Infiltrated capillary structure		
E	VEGF 20 ng/mL	SD Rat aorta ring (ex vivo)	10~20 μg/mL	↓Sprouts from aortic rings		
E	VEGF 150 ng/mL	Male C57BL/6 mice (in vivo)	10~20 μg/mL for 6d	↓Blood vessels infiltrated into Matrigel plug		
E	RC-58T/h/SA#4 cells	Male Balb/c mice (in vivo)	25, 50 mg/kg	↓Blood vessels infiltrated into tumor	N.A.	[52]
W	None	HepG2 cells (in vitro)	25 μg/mL	↓VEGF, bFGF mRNA, protein ↓Extracellular secretion of VEGF, bFGF	↓p-Akt protein ↓ p-ERK protein	[67]

E: ethanol extract; M: methanol extract; W: water extract; EA; ethyl acetate fraction; VEGF: vascular endothelial growth factor; HUVECs: human umbilical vein endothelial cells; bFGF: basic fibroblast growth factor; FAK: focal adhesion kinase; PI3K: phosphoinositide 3-kinase; Akt: protein kinase B; mTOR: mammalian target of rapamycin; ERK: extracellular-signal-regulated kinase. “↓” and “↑” represent the decrease or increase of the value.

**Table 3:** Antimetastatic effects and molecular mechanisms of *O. japonica.*

Extract	Inducer	Model	Concentration	Result	Mechanism	Ref
EA	None	MDA-MB-231 (in vitro)	20, 40, 60 μg/mL	↓Wound closure area	↓MMP-9 protein	[41]
M	PMA	THP-1 (in vitro)	10, 25 μg/mL	↓Adherent cells	↓MMP-2/9 mRNA ↓p-p38, p-ERK protein ↓NF-κB p65 protein	[73]
E	None	HT1080 (in vitro)	200, 300 μg/mL	↓Migrated cells	↓MMP-2/9 protein	[74]
M	None	LNCaP (in vitro)	200 μg/mL	↓Wound closure area ↓Migrated cells	↓MMP-2/9 mRNA/protein ↑TIMP-1/2 protein ↓Claudin-1/3 mRNA ↓p-Akt prtoein	[75]
E	Hypoxia	RC-58T/h/SA#4 (in vitro)	50, 100 μg/mL	↓Wound closure area ↓Migrated cells	↓HIF-1α protein ↓p-FAK protein ↓uPA protein ↓p-ERK1/2 protein ↓PI3K/Akt protein	[52]
M	Collagen I/IV	A549 (in vitro)	400 μg/mL	↓Adherent cells	N.A	[76]
	Laminin	B16-F0 (in vitro)	400 μg/mL	↓Adherent cells		

E: ethanol extract; M: methanol extract; W: water extract; EA; ethyl acetate fraction; PMA: phorbol 12-myristate 13-acetate; HIF: hypoxia-inducible factors; uPA: urokinase-type plasminogen activator; MMP: matrix metalloproteinases; TIMP: tissue inhibitors of metalloproteinases; FAK: focal adhesion kinase; PI3K: phosphoinositide 3-kinase; Akt: protein kinase B; ERK: extracellular-signal-regulated kinase. “↓” and “↑” represent the decrease or increase of the value.

## Data Availability

Data sharing is not applicable to this article.
